# Hospital-based surveillance for Japanese encephalitis in Bangladesh, 2007–2016: Implications for introduction of immunization

**DOI:** 10.1016/j.ijid.2020.07.026

**Published:** 2020-10

**Authors:** Kishor Kumar Paul, Hossain M.S. Sazzad, Mahmudur Rahman, Sharmin Sultana, M. Jahangir Hossain, Jeremy P. Ledermann, Paul Burns, Michael S. Friedman, Meerjady S. Flora, Marc Fischer, Susan Hills, Stephen P. Luby, Emily S. Gurley

**Affiliations:** a International Centre for Diarrhoeal Disease Research, Bangladesh (icddr,b), Dhaka, Bangladesh; b The Kirby Institute, University of New South Wales, Sydney, Australia; c Institute of Epidemiology, Disease Control and Research (IEDCR), Dhaka, Bangladesh; d Medical Research Council Unit The Gambia at the London School of Hygiene & Tropical Medicine, Banjul, Gambia; e Arboviral Diseases Branch, Centers for Disease Control and Prevention (CDC), Fort Collins, CO, USA; f CDC, Country Office for Bangladesh, Dhaka, Bangladesh; g Stanford University, Stanford, CA, USA; h Johns Hopkins Bloomberg School of Public Health, Baltimore, MD, USA

**Keywords:** Japanese encephalitis, Vaccine-preventable disease, Hospital-based surveillance, Causes of encephalitis, Vaccination

## Abstract

**Background:**

Japanese encephalitis (JE) virus is recognized as a major cause of encephalitis in Bangladesh. The World Health Organization (WHO) recommends human immunization as the most effective means to control JE. Several WHO-prequalified vaccines are available to prevent JE but no vaccination program has been implemented in Bangladesh.

**Methods:**

We conducted hospital-based surveillance for acute meningitis-encephalitis syndrome (AMES) to describe JE epidemiology and help inform policy decisions about possible immunization strategies for Bangladesh.

**Results:**

During 2007–2016, a total of 6543 AMES patients were identified at four tertiary hospitals. Of the 6525 patients tested, 548 (8%) were classified as JE cases. These 548 patients resided in 36 (56%) out of 64 districts of Bangladesh, with the highest proportion of JE cases among AMES patients (12% and 7%) presenting at two hospitals in the northwestern part of the country. The median age of JE cases was 30 years, and 193 (35%) were aged ≤15 years. The majority of JE cases (80%) were identified from July through November.

**Conclusions:**

Surveillance results suggest that JE continues to be an important cause of meningo-encephalitis in Bangladesh. Immunization strategies including JE vaccine introduction into the routine childhood immunization program or mass vaccination in certain age groups or geographic areas need to be examined, taking into consideration the cost-effectiveness ratio of the approach and potential for decreasing disease burden.

## Highlights

• Japanese encephalitis (JE) is largely preventable through vaccination.
• Several JE vaccines prequalified by World Health Organization are available.
• Hospital-based surveillance were conducted in Bangladesh to describe JE epidemiology.
• JE cases were identified each year, among all age groups, and from a widespread geographical area.
• Routine childhood immunization program or mass vaccination need to be examined.

## Introduction

Japanese encephalitis virus (JEV), a mosquito-borne flavivirus, primarily causes asymptomatic infection in humans, but can also cause illness ranging from fever and headache to severe encephalitis (Halstead and Jacobson, 2003). About 3 billion people in 25 countries in Asia and the western Pacific are at risk of Japanese encephalitis (JE). While there are no recent estimates of global JE incidence, in 2011 a systematic review estimated 67,900 clinical cases of JE typically occurred annually (Campbell et al., 2011). Approximately 20–30% of cases are fatal and 30–50% of survivors have neurologic or psychiatric sequelae (Campbell et al., 2011). The first JE outbreak reported from Bangladesh occurred in 1977 in the central part of the country (Khan et al., 1981). A study to assess etiologies of encephalitis in Bangladesh, including JE, was conducted in four tertiary care hospitals during 2003–2005 and provided evidence that JEV was the most common identifiable cause of viral encephalitis (Hossain et al., 2010). A subsequent study found the highest incidence of JE in northwest Bangladesh, estimated at 2.7/100,000 population in Rajshahi (Paul et al., 2011).

JEV is maintained in an enzootic transmission cycle between *Culex* mosquitoes and animal hosts, mostly pigs and wading birds; humans are infected incidentally and do not transmit the virus to others. Although no effective antiviral therapy is available, JE is largely preventable through vaccination and many countries in Asia have reduced JE incidence by vaccine introduction into their national immunization programs (Ginsburg et al., 2017, Heffelfinger et al., 2017, Wu et al., 1999). The World Health Organization (WHO) has prequalified several JE vaccines and recommends them for use in routine immunization programs where the burden of disease warrants intervention (WHO, 2016). Bangladesh has not yet introduced JE vaccine into the national immunization program. International Centre for Diarrhoeal Disease Research, Bangladesh (icddr,b) in collaboration with Institute of Epidemiology, Disease Control and Research (IEDCR), Ministry of Health and Family Welfare, Government of Bangladesh conducted hospital-based surveillance during September 2007–July 2016 to assess JE epidemiology to inform public health decision-making about vaccine introduction.

## Methods

### Surveillance sites

**Figure 1:**
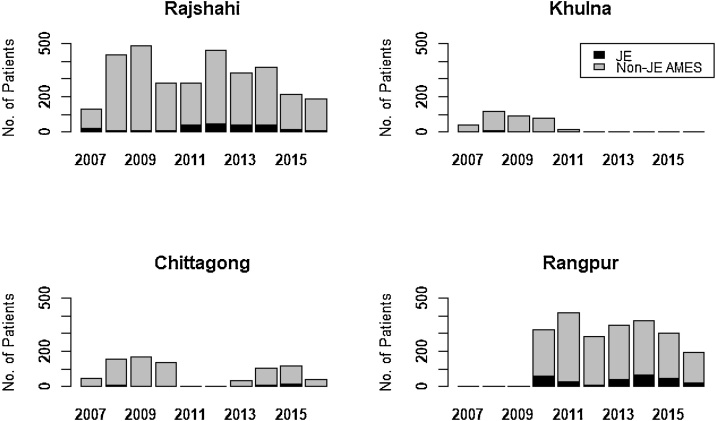
Number of acute meningitis-encephalitis syndrome (AMES) patients classified as Japanese encephalitis (JE) and non-JE identified at four surveillance sites across Bangladesh, 2007–2016. Surveillance was not conducted in Chittagong, Rangpur, and Khulna for the periods 2011–2012, 2007–2009 and 2012–2016 respectively.

**Figure 2:**
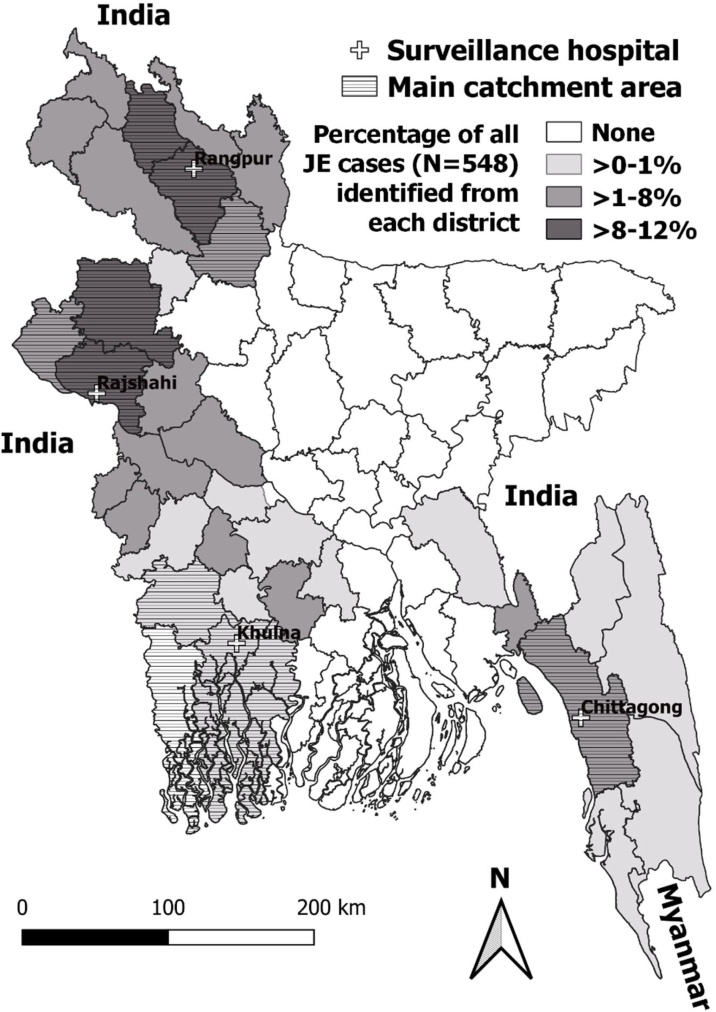
Japanese encephalitis surveillance sites, main catchment areas, and case locations in Bangladesh, 2007–2016.

In September 2007, acute meningitis-encephalitis syndrome (AMES) surveillance was initiated in three tertiary care referral hospitals (Rajshahi, Khulna, and Chittagong Medical College Hospitals); in January 2010, a fourth site (Rangpur Medical College Hospital) was included (Figure 1, Figure 2 and Supplementary Table). Surveillance activities varied over time due to funding constraints: enrolment in Khulna Medical College Hospital was discontinued at the end of 2010, and no surveillance was conducted in Chittagong Medical College Hospital during the years 2011 and 2012.

### AMES surveillance case definition and collection of patient data and samples

For the purposes of surveillance, we defined a case of AMES as a patient who had acute onset of measured or history of fever (≥38 °C) and one or more of the following within five days prior to hospitalization: 1) altered mental status (confusion, lethargy, agitation, coma), 2) a neurologic deficit (focal or diffuse neurological dysfunction involving motor and cranial nerves, or new onset of seizures), or 3) signs of meningeal irritation (neck rigidity or positive Kernig's sign). Surveillance physicians visited adult medicine and pediatrics wards daily except Fridays to identify patients meeting the AMES clinical case definition, collect demographic and clinical information using a standardized form, and coordinate collection of cerebrospinal fluid (CSF) and two samples of serum, one at admission and another at discharge. In case of a patient's death before sample collection, data were collected from hospital registers or caregivers.

### JE laboratory testing and JE case definition

The specimens were divided into aliquots at the sentinel sites, stored in liquid nitrogen dry shippers, and transported weekly to the IEDCR, Dhaka for laboratory testing for JEV infection and subsequent storage at − 70 °C. At IEDCR, the CSF and serum samples were tested for anti-JEV immunoglobulin M (IgM) antibodies using the Panbio JE-Dengue IgM combo enzyme-linked immunosorbent assay (ELISA) up to April 2014, and later using the InBios JE Detect Capture ELISA and DENV Detect Capture ELISA (Johnson et al., 2016). Samples were shipped on dry ice to the U.S. Centers for Disease Control and Prevention (CDC) Arboviral Diseases Branch, Fort Collins for confirmatory testing, initially using a JE IgM capture ELISA to test CSF and serum samples (Martin et al., 2000). Serum specimens positive or equivocal for IgM against JEV were tested for JEV and dengue virus 1 and 2 neutralizing antibodies by plaque reduction neutralization test (PRNT) using an 80% cut-off value (80% PRNT). Based on this testing, a JE case was defined as a patient meeting the AMES clinical definition with 1) IgM against JEV in CSF, or 2) IgM against JEV in serum with a JEV 80% PRNT titer ≥ 20 and a JEV to dengue virus neutralizing antibody titer ratio ≥4.

### Ethical considerations

All procedures and testing were consistent with routine clinical practices. However, because the activity took place in a clinical setting and the results were not provided in a way that would inform clinical care, surveillance physicians took informed written consent for data and sample collection from the patient before enrolment. If the patient was unable to provide consent, or if the patient was a child, consent was obtained from the patient's family member or guardian. The protocol was reviewed and approved by the ethics committee at icddr,b and determined to be a non-research surveillance activity after human subjects review at CDC.

## Results

From the start of the surveillance at each of the four sentinel hospitals through July 2016, a total of 6543 AMES cases were identified. Among the cases, 3167 were identified at Rajshahi, 2242 at Rangpur, 793 at Chittagong, and 341 at Khulna. The median age of AMES patients was 15 years with interquartile range (IQR) 4–38 years; however, the median age varied from 5 years at Chittagong to 20 years at Rangpur. Overall, 4055 (62%) of the AMES cases were male. Among the 6543 patients, 6525 (99.7%) consented to be enrolled and provided at least one serum or CSF specimen for JE testing.

### Demographics of JE cases

Overall, 548 (8%) of the 6525 tested patients had laboratory evidence of recent JEV infection, including 12% (263 of 2236) of patients at Rangpur, 7% (229 of 3162) at Rajshahi, 5% (42 of 792) at Chittagong, and 4% (14 of 335) at Khulna (Table 1). An additional 88 AMES patients were initially diagnosed as JE at IEDCR, but following confirmatory testing at CDC they were subsequently classified as having dengue infection (9), unspecified flaviviruses infection (23), or no JEV infection (56).

**Table 1:** Features of Japanese encephalitis cases at each surveillance hospital in Bangladesh, 2007–2016

Characteristics	Rangpur	Rajshahi	Chittagong	Khulna	TOTAL
	(*N* = 263) *n* (%)	(*N* = 229) *n* (%)	(*N* = 42) *n* (%)	(*N* = 14) *n* (%)	(*N* = 548) *n* (%)
*Age (years)*					
≤5	30 (11)	25 (11)	12 (29)	–	67 (12)
6–15	57 (22)	50 (22)	15 (36)	4 (29)	126 (23)
16–25	30 (11)	27 (12)	5 (12)	–	62 (11)
26–35	25 (10)	19 (8)	3 (7)	2 (14)	49 (9)
36–45	27 (10)	19 (8)	1 (2)	2 (14)	49 (9)
46–55	32 (12)	31 (14)	5 (12)	2 (14)	70 (13)
56–65	39 (15)	41 (18)	–	2 (14)	82 (15)
≥66	23 (9)	17 (7)	1 (2)	2 (14)	43 (8)
*Sex*					
Male	162 (62)	146 (64)	23 (55)	10 (71)	341 (62)
Female	101 (38)	83 (36)	19 (45)	4 (29)	207 (38)
*Month*					
Jan–Mar	10 (4)	5 (2)	1 (2)	–	16 (3)
Apr–Jun	48 (18)	15 (7)	15 (36)	3 (22)	81 (15)
Jul–Sep	133 (51)	83 (36)	9 (21)	2 (14)	227 (41)
Oct–Dec	72 (27)	126 (55)	17 (41)	9 (64)	224 (41)
*Year*					
2007^a^	–	20 (9)	2 (5)	2 (14)	24 (4)
2008	–	11 (5)	10 (24)	5 (36)	26 (5)
2009	–	10 (4)	2 (5)	–	12 (2)
2010	60 (23)	10 (4)	3 (7)	3 (21)	76 (14)
2011	26 (10)	39 (17)	–	4 (29)	69 (12)
2012	9 (3)	45 (20)	–	–	54 (10)
2013	39 (15)	38 (17)	4 (10)	–	81 (15)
2014	67 (25)	37 (17)	5 (12)	–	109 (20)
2015	44 (17)	14 (6)	12 (29)	–	70 (13)
2016^b^	18 (7)	5 (2)	4 (10)	–	27 (5)

a Started in September 2007.

b As of July 2016.

**Figure 3:**
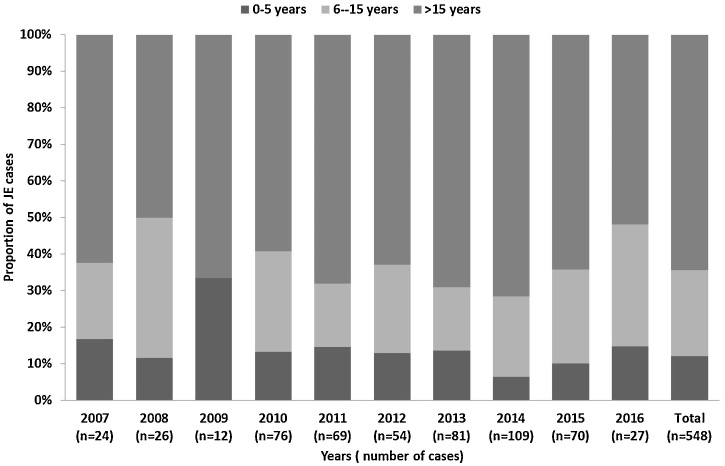
Age group distribution of Japanese encephalitis cases, Bangladesh, 2007–2016.

In total, 341 (62% of 548) JE cases were male. The median age of JE cases was 30 years (IQR 11–55 years); median age varied by location, ranging from 11 years at Chittagong to 40 years at Khulna. Overall, 193 (35%) JE cases were aged ≤15 years, ranging from 64% (27 of 42) at Chittagong to 29% (4 of 14) at Khulna. During 2007 through 2016, the percentage of JE patients aged ≤15 years ranged from 28–50% (Figure 3). The proportion of AMES cases classified as JE was lowest in children aged ≤5 years (3% [67 of 1925]) and was highest in patients aged 56–65 years (20% [82 of 406]).

### Temporo-spatial distribution of JE cases

**Figure 4:**
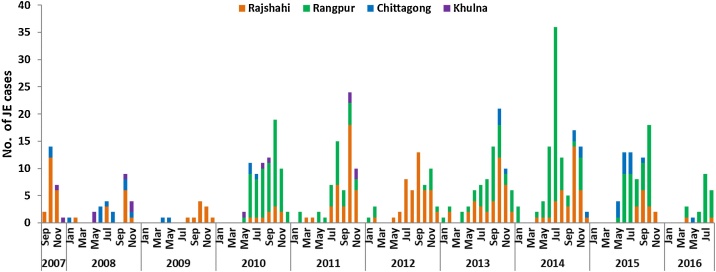
Number of Japanese encephalitis cases identified by month of symptom onset from Rajshahi, Rangpur, Chittagong, and Khulna Medical College Hospitals, Bangladesh, September 2007–July 2016 (*N* = 548).

A seasonal increase in JE cases was observed starting in the second quarter of each year and cases usually peaked during the month of October (Figure 4). Overall, 436 (80%) cases had illness onset from July through November. While the changing number of sentinel sites included in surveillance each year affected the annual number of JE cases, the highest number of detected JE cases in one calendar year (n = 109) was in 2014, including 67 cases from Rangpur. The lower number of total reported cases in 2007–2009 is explained by enrolment at Rangpur starting only in 2010. The proportion of AMES cases classified as JE ranged from 2% (12 of 746) in 2009 to 13% (109 of 834) in 2014. The cases identified in surveillance hospitals resided in 36 (56%) of 64 districts of Bangladesh (Figure 2). The percentage of all JE cases identified from each district ranged from 0.2% in Jessore, Comilla, and Bagerhat districts to 12.1% in Rangpur district.

## Discussion

Long-term AMES surveillance among patients admitted to four sentinel hospitals in Bangladesh identified recent JEV infection in 548 (8%) of 6525 AMES patients tested during September 2007 through July 2016. JE cases were reported in each year, among all age groups, and from a widespread geographical area. These results indicate JEV is an ongoing and important cause of encephalitis in Bangladesh.

In previous hospital-based surveillance conducted from 2003–2005 at four tertiary care hospitals in Dhaka, Mymensingh, Sylhet and Rajshahi, JEV was recognized as the most common identifiable cause of infection among patients presenting with acute encephalitis (Hossain et al., 2010). The current surveillance, again conducted at Rajshahi Medical College, as well as at three other medical colleges in Rangpur, Chittagong, and Khulna, expanded the area of recognized JEV transmission. While patients residing further from one of the four sentinel sites were probably less likely to be admitted to one of the surveillance hospitals, nonetheless JE patients resided in 56% of Bangladesh's 64 districts. Combined results of the surveillance activities during 2003–2005 and 2007–2016 show JEV infections have been detected among residents of almost every district of Bangladesh. There is no evidence that JEV transmission has been interrupted since the earlier surveillance activities, confirming JE endemicity in Bangladesh.

JE cases were identified in all four surveillance hospitals; however, JEV seropositivity was higher in the Rangpur and Rajshahi sites compared with the other two surveillance hospitals. This finding in Rajshahi is consistent with a previous survey that determined JE incidence to be higher in Rajshahi than in Khulna or Chittagong (Paul et al., 2011). This is possibly associated with a higher concentration of pigs, a major vertebrate host in the JEV transmission cycle, in the northwestern areas of Bangladesh. A comprehensive pig census conducted in Rajshahi, Nawabganj, and Naogaon districts, the primary catchment area of Rajshahi hospital, estimated 50% of pigs were infected with JEV by 3 years of age with increasing seroprevalence levels according to age (Khan et al., 2014). However, no published pig distribution data were available from the catchment areas of the other sentinel hospitals. In addition, *Culex tritaeniorhynchus* mosquitoes, the main vectors of JEV, breed in wet paddy fields (Innis, 1996), and rice production, as well as the proportion of people directly involved in rice cultivation, is higher in this part of the country than in any other area (Bangladesh Bureau of Statistics, 2010). Wading birds, another known vertebrate host of JEV, are also frequently found in paddy fields (Maeda, 2001). Furthermore, ducks and chicken are able to produce sufficient enough viremia if infected at an early age to act as amplifying hosts (Cleton et al., 2014) and their absolute number and density in Bangladesh is high (Bangladesh Bureau of Statistics, 2019).

In the previous population-based JE incidence survey in Bangladesh in 2008–2009, estimated JE incidence was 0.6/100,000 population in Chittagong, 1.4 in Khulna, and 2.7 in Rajshahi (Paul et al., 2011). Estimates were based on the proportion of JE cases among AMES cases which was 6–7% at the three locations. While population-based JE incidence could not be determined from the current hospital-based surveillance, a similar proportion (8%) of AMES cases admitted to sentinel hospitals during the current surveillance period had evidence of recent JEV infection. Given this proportion remains similar to the earlier study, there is little reason to believe incidence has decreased. Similar JE incidences (Paul et al., 2011) were reported in some other JE endemic countries before introduction of JE vaccine into the national immunization programs in those countries (Endy and Nisalak, 2002, Wierzba et al., 2008, Wu et al., 1999).

Most JE cases (80%) occurred during July–November, corresponding to the monsoon and post-monsoon months. This is likely related to the abundance of *Cx. tritaeniorhynchus* in these months, due to prolific breeding in saturated paddy fields and in other collections of water (Upadhyayula et al., 2012).

JE is considered a childhood disease in most JE-endemic countries, with about 75% of all annual JE cases occurring in children aged 0–14 years (Campbell et al., 2011). In Bangladesh, both children and adults are affected by JE. The median age of JE cases was 30 years, and at three out of four sites about two-thirds of cases were in persons aged >15 years; only in Chittagong were the majority (64%) of cases aged ≤15 years. This was similar to the previous hospital-based surveillance in 2003–2005, in which 55% cases were >15 years of age (Hossain et al., 2010). This might reflect a lower force of infection in Bangladesh than in many other endemic countries, with more persons entering adulthood without having been exposed to JEV and thus remaining susceptible to infection, or more recent introduction of JEV. Despite this unusual epidemiologic pattern, there is still a relatively high impact of JE among children with 35% cases aged ≤15 years.

In JE endemic settings, WHO recommends a one-time “catch-up” campaign followed by incorporation of JE vaccine into the routine childhood immunization program (WHO, 2016). Countries are encouraged to adopt immunization strategies based on local epidemiology and feasibility. For example, in JE-endemic districts of India, children from 1 to 15 years of age were vaccinated by large vaccination campaigns in a phased manner followed by introduction of JE vaccine into the routine immunization program [17]. Following these vaccination campaigns, adult JE cases outnumbered pediatric cases in some JE endemic states which led some of the state governments to conduct special JE vaccination campaigns in adults aged > 15 years (Medhi et al., 2017, Vashishtha and Ramachandran, 2015). Considering the median age of JE cases in Bangladesh is 30 years, introduction of a childhood vaccination program would have an important impact on reducing JE cases, but reductions in adult cases would take years to accomplish, and would only be observed as the vaccinated cohort aged into adulthood unless special campaigns targeting adults were also undertaken. Currently, Gavi Alliance provides funding support for JE vaccination campaigns for children aged 9 months–14 years. In Bangladesh, a campaign in this age group would prevent about one-third of JE cases. While not an ideal strategy, it could be considered a useful step towards JE control as it would likely provide lifelong protection to these children, and a higher coverage rate would probably be achieved compared with a full population campaign, given the greater feasibility of vaccine delivery to children and adolescents.

We note certain limitations of this surveillance. The surveillance platform is likely to underestimate the true number of JE cases. If surveillance had been ongoing in all hospitals without interruption, the number of detected JE cases would have been higher. In addition, many patients with AMES never reach surveillance hospitals due to the long distance from their residence or unwillingness to seek healthcare (Ahmed et al., 2001, Syed et al., 2008).

Since there is no specific treatment for JE, control strategies could include immunization of humans and animals, and vector control. However, in the context of limited evidence regarding the effectiveness of vector control, bed nets, swine immunization or animal sequestration (Igarashi, 2002), WHO recommends that countries achieve and maintain high human vaccine coverage to realize the greatest reductions in the number of human JE cases (WHO, 2016). Studies conducted in other JE-endemic Asian countries have reported human immunization is effective in terms of decreasing AMES burden and health care costs (Touch et al., 2010, Upreti et al., 2017, Yin et al., 2012). Immunization strategies including but not limited to introduction of JE vaccine into the routine childhood immunization program, mass vaccination of children under 15 years of age, and mass vaccination in limited geographic areas need to be examined, considering the cost-effectiveness ratio of the approach and potential for substantially decreasing disease burden.

## Funding

This work was supported by Centers for Disease Control and Prevention (CDC), USA [cooperative agreement no: 5U01CI000628]; this financial support was in part from a grant to CDC provided by The Bill & Melinda Gates Foundation (grant #OPPGH5333). In addition, the Government of Bangladesh, Canada, Sweden and the UK provided core/unrestricted funding support for this work.

## Conflict of interest

The authors have no competing interests to declare.

## Disclaimer

The findings and conclusions in this report are those of the authors and do not necessarily represent the official position of CDC.
