# Supplementary material for: Hospital-based surveillance for Japanese encephalitis in Bangladesh, 2007–2016: Implications for introduction of immunization
Source: Int J Infect Dis. 2020 Oct;99:69–74. doi: 10.1016/j.ijid.2020.07.026 (PMC7566160; doi:10.1016/j.ijid.2020.07.026)
Supplement: Supplementary file 1 [file mmc1.docx]

**Supporting Information**

S1 Table: Features of Japanese encephalitis (JE) and Acute Meningitis-Encephalitis Syndrome (AMES) cases at each surveillance hospital in Bangladesh, 2007–2016

|  | **Rangpur** | | | **Rajshahi** | | | **Chittagong** | | | **Khulna** | | | **TOTAL** | | |
| --- | --- | --- | --- | --- | --- | --- | --- | --- | --- | --- | --- | --- | --- | --- | --- |
| **Characteristics** | **JE** | **AMES** | **(%)** | **JE** | **AMES** | **(%)** | **JE** | **AMES** | **(%)** | **JE** | **AMES** | **(%)** | **JE** | **AMES** | **(%)** |
|  | n=263 | n=2,236 | (12) | n=229 | n=3,162 | (7) | n=42 | n=792 | (5) | n=14 | n=335 | (4) | N=548 | N=6,525 | (8) |
| **Age (years)** |  |  |  |  |  |  |  |  |  |  |  |  |  |  |  |
| ≤5 | 30 | 433 | (7%) | 25 | 963 | (3) | 12 | 403 | (3) | 0 | 126 | (0) | 67 | 1,925 | (3) |
| 6–15 | 57 | 482 | (12) | 50 | 626 | (8) | 15 | 161 | (9) | 4 | 110 | (4) | 126 | 1,379 | (9) |
| 16–25 | 30 | 400 | (8) | 27 | 453 | (6) | 5 | 75 | (7) | 0 | 26 | (0) | 62 | 954 | (7) |
| 26–35 | 25 | 236 | (11) | 19 | 289 | (7) | 3 | 45 | (7) | 2 | 19 | (11) | 49 | 589 | (8) |
| 36–45 | 27 | 180 | (15) | 19 | 221 | (9) | 1 | 32 | (3) | 2 | 13 | (15) | 49 | 446 | (11) |
| 46–55 | 32 | 205 | (16) | 31 | 235 | (13) | 5 | 40 | (13) | 2 | 10 | (20) | 70 | 490 | (14) |
| 56–65 | 39 | 166 | (23) | 41 | 203 | (20) | 0 | 20 | (0) | 2 | 17 | (12) | 82 | 406 | (20) |
| ≥66 | 23 | 134 | (17) | 17 | 172 | (10) | 1 | 16 | (6) | 2 | 14 | (14) | 43 | 336 | (13) |
| **Sex** |  |  |  |  |  |  |  |  |  |  |  |  |  |  |  |
| Male | 162 | 1348 | (12) | 146 | 2,012 | (7) | 23 | 476 | (5) | 10 | 206 | (5) | 341 | 4,042 | (8) |
| Female | 101 | 888 | (11) | 83 | 1,150 | (7) | 19 | 316 | (6) | 4 | 129 | (3) | 207 | 2,483 | (8) |
| **Month** |  |  |  |  |  |  |  |  |  |  |  |  |  |  |  |
| Jan–Mar | 10 | 392 | (3) | 5 | 558 | (1) | 1 | 159 | (1) | 0 | 75 | (0) | 16 | 1184 | (1) |
| Apr–Jun | 48 | 714 | (7) | 15 | 792 | (2) | 15 | 243 | (6) | 3 | 84 | (4) | 81 | 1833 | (4) |
| Jul–Sep | 133 | 614 | (22) | 83 | 972 | (9) | 9 | 151 | (6) | 2 | 84 | (2) | 227 | 1821 | (12) |
| Oct–Dec | 72 | 516 | (14) | 126 | 840 | (15) | 17 | 239 | (7) | 9 | 92 | (10) | 224 | 1687 | (13) |
| **Year** |  |  |  |  |  |  |  |  |  |  |  |  |  |  |  |
| 2007^a^ | - | - | - | 20 | 130 | (15) | 2 | 44 | (5) | 2 | 39 | (5) | 24 | 213 | (11) |
| 2008 | - | - | - | 11 | 434 | (3) | 10 | 153 | (7) | 5 | 114 | (4) | 26 | 701 | (4) |
| 2009 | - | - | - | 10 | 485 | (2) | 2 | 168 | (1) | 0 | 93 | (0) | 12 | 746 | (2) |
| 2010 | 60 | 323 | (19) | 10 | 278 | (4) | 3 | 134 | (2) | 3 | 77 | (4) | 76 | 812 | (9) |
| 2011 | 26 | 418 | (6) | 39 | 278 | (14) | - | - | - | 4 | 12 | (33) | 69 | 708 | (10) |
| 2012 | 9 | 285 | (3) | 45 | 463 | (10) | - | - | - | - | - | - | 54 | 750 | (7) |
| 2013 | 39 | 345 | (11) | 38 | 335 | (11) | 4 | 36 | (12) | - | - | - | 81 | 714 | (11) |
| 2014 | 67 | 371 | (18) | 37 | 362 | (10) | 5 | 101 | (5) | - | - | - | 109 | 834 | (13) |
| 2015 | 44 | 299 | (15) | 14 | 210 | (7) | 12 | 115 | (10) | - | - | - | 70 | 624 | (11) |
| 2016^b^ | 18 | 194 | (9) | 5 | 187 | (3) | 4 | 41 | (10) | - | - | - | 27 | 422 | (6) |

^a^started in September 2007

^b^as of July 2016
